# Impact of active lifestyle on the primary school children saliva microbiota composition

**DOI:** 10.3389/fnut.2023.1226891

**Published:** 2023-08-10

**Authors:** Annamaria Mancini, Claudia Cerulli, Daniela Vitucci, Vito Alessandro Lasorsa, Daniela Parente, Andrea Di Credico, Stefania Orrù, Paolo Riccardo Brustio, Corrado Lupo, Alberto Rainoldi, Federico Schena, Mario Capasso, Pasqualina Buono

**Affiliations:** ^1^Department of Movement Sciences and Wellness, University Parthenope, Naples, Italy; ^2^CEINGE-Biotecnologie Avanzate “Franco Salvatore”, Napoli, Italy; ^3^Department of Movement, Human and Health Sciences, University of Rome "Foro Italico", Rome, Italy; ^4^Department of Molecular Medicine and Medical Biotechnologies, University of Naples Federico II, Naples, Italy; ^5^Reprogramming and Cell Differentiation Lab, Center for Advanced Studies and Technology (CAST), Chieti, Italy; ^6^Department of Medicine and Aging Sciences, University "G. D'Annunzio" of Chieti-Pescara, Chieti, Italy; ^7^Department of Clinical and Biological Sciences, University of Torino, Turin, Italy; ^8^Department of Medical Sciences, University of Torino, Turin, Italy; ^9^Department of Neuroscience, Biomedicine and Movement, University of Verona, Verona, Italy

**Keywords:** lifestyle, exercise, saliva, microbiota, children

## Abstract

The aim of the study was to evaluate the effects of Active or Sedentary lifestyle on saliva microbiota composition in Italian schoolchildren.

**Methods:**

Male (114) and female children (8–10 years) belonging to five primary schools in the neighborhoods of Turin were classified as active (A) or sedentary (S) based on PAQ-C-It questionnaire. PCR amplification of salivary DNA targeted the hypervariable V3–V4 regions of the 16S rRNA bacterial genes. DADA2 workflow was used to infer the Amplicon Sequence Variants and the taxonomic assignments; the beta-diversity was obtained by PCoA with the UniFrac method; LEfSe algorithm, threshold at 5%, and Log LDA cutoff at ±0.5 were used to identify differently abundant species in A compared to S saliva sample. Daily food intake was assessed by 3-Days food record. The metabolic potential of microbial communities was assessed by PICRUSt.

**Results:**

No significant differences were found in individual’s gender distribution (*p* = 0.411), anthropometry, BMI (*p* > 0.05), and all diet composition between A and S groups (*p* > 0.05). Eight species were differently abundant: *Prevotella nigrescens* (LDA score = −3.76; FDR = 1.5×10–03), *Collinsella aerofaciens* (LDA score = −3.17; FDR = 7.45×10–03), *Simonsiella muelleri* (LDA score = −2.96; FDR = 2.76×10–05), *Parabacteroides merdae* (LDA score = −2.43; FDR = 1.3×10–02) are enriched in the A group; *Gemella parahaemolysans*, *Prevotella aurantiaca* (LDA score = −3.9; FDR = 5.27×10–04), *Prevotella pallens* (LDA score = 4.23; FDR = 1.93×10–02), *Neisseria mucosa* (LDA score = 4.43; FDR = 1.31×10–02; LDA score = 2.94; FDR = 7.45×10–03) are enriched in the S group. A prevalence of superpathway of fatty acid biosynthesis initiation (*E. coli*) and catechol degradation II (meta-cleavage pathway) was found in saliva from A compared to S children.

**Conclusion:**

Our results showed that active children had an enrichment of species and genera mainly associated with a healthier profile. By contrast, the genera and the species enriched in the sedentary group could be linked to human diseases.

## 1. Introduction

The human gut microbiota is intimately associated with different aspects of human health and disease. Its characterization could help diagnosis, prognosis, and therapy settings by giving over 150 times more genetic information than the human genome alone (1). The microbiota composition depends on spatial distribution and age; in general, the microbiota diversity increases over the time and decreases in elderly (2, 3). In children of about 3 years, gut microbiota becomes similar to that of adults, with five predominant bacterial phyla: Firmicutes, Bacteroidetes, Actinobacteria, Proteobacteria and Verrucomicrobia (4). Recent findings established the role of environmental factors on microbiota composition suggesting a close cross-talk between the lifestyle and the diversity of microorganisms populating the intestine (5). To date, the effects of exercise on human health have been thoroughly studied. In the last decade, many evidences supported a dynamic relationship between the composition of gut microbiota (GM) and physical activity levels in animal models (6–10) and in human (11–14). In particular, the theory that exercise is able to enrich the diversity of the human gut microbiota depending on the volume of training resulting in improved health status of the host, has been supported (12, 15). In particular, GM diversity has been associated to cardiorespiratory fitness (11, 16, 17) and to VO2max in adults (18). Moreover, Barton et al. (18) highlighted in GM, by a metagenomic and metabolomic approach, relative increases in fecal pathways and metabolites, e.g., short-chain fatty acids (SCFAs) produced by microbes, associated with higher muscle turnover and overall health in professional athletes compared with sedentary controls.

While in adults there are some evidences of the influence of physical activity on GM composition, in children or pre-adolescents, very poor results have been provided. Recent reports indicated that the GM profile is associated with the body mass index and could be modulated by exercise training and lifestyle habits in obese children (19–22). Furthermore, several evidences show that the salivary microbiota mirrors the gut microbiota and that some oral bacteria colonize the gut and have been associated both to oral and systemic health. (23–26).

Despite these recent advances, the complete landscape of the association between the saliva profile and lifestyle habits in children is still to be clarified. Further, no data on saliva microbiota composition have been provided in Italian schoolchildren associated to Active compared to Sedentary status, to date. Thus, the principal aim of this study was to analyse the possible association between saliva microbiota compositions and lifestyle in Active compared to Sedentary cohort of 8–10-year-old Italian school-aged children living in the neighborhoods of Turin (northwest Italy).

We conducted this study by hypothesizing that active lifestyle could be associated with saliva microbiota profiles contributing to host health promotion. Indeed, the main aim of our work was to identify the differences in the saliva of Active compared to Sedentary schoolchildren. In order to study the microbiota composition, we sequenced the bacterial 16S rRNA of saliva biospecimens and assessed their differential abundance.

## 2. Materials and methods

### 2.1. Participants

One hundred and thirty children (8–10 years) belonging to five primary schools in the neighborhoods of Turin (northwest Italy) were enrolled. All information on the aim of the study has been provided to children’s parents/guardians and teachers as previously described (27).

Children meeting any of the following criteria were excluded from the study: (i) recent infections (1 month prior to sample collection), (ii) having disorders affecting diet or physical activity, and (iii) recent usage of either antibiotic, prebiotics and probiotics supplements (1 month prior to data and sample collection). The enrolled children were classified in two groups: active (A) and sedentary (S) on the basis of Physical Activity Questionnaire for Older Children (PAQ-C-It cut-off score of 2.75), the related procedures are detailed in Lupo et al. (27). Parents/guardians and teachers provided written informed consent for participation to the study, according to the ethical standards provided in the 1964 Declaration of Helsinki. Ethics committee on human research of the University of Turin (9 March 2020: Protocol #134691) and Naples (17 January 2020: Protocol #376/19) approved the study. The procedures used to take anthropometric measures were described in Lupo et al. (27); briefly, stature was measured by a portable stadiometer (Model 214; Seca, Hamburg, Germany), body mass was measured by an electronic scale (Model 876; Seca, Hamburg, Germany), participants’ waist circumference was measured in the standing position, midway between the lowest rib and the iliac crest by Ana elastic meter. The Body Mass Index (BMI) was calculated as body mass divided by height squared (kg/m^2^).

To estimate the daily food intake, all participants filled the questionnaire (3-Days food records). Records were processed using Winfood software (Medimatica S.u.r.l., Colonnella, TE, Italy). Statistical analysis was performed through a one-way ANOVA (Statview software).

### 2.2. Saliva sample collection and genomic DNA extraction

The donor was asked not to eat and not to use oral hygiene products 1 h before saliva collection. At least 2 mL of unstimulated saliva was collected, put on ice and stored at − 80°C until the analysis. DNA was extracted from saliva samples using the MagPurix Bacterial DNA Extraction Kit (ZP02006; Zinexts Life Science Corp.) according to the manufacturer’s instructions. DNA was quantified using the Qubit dsDNA BR and HS assay kit (Life Technologies, CA, United States).

### 2.3. Preparation of the 16S metagenomic sequencing library

PCR amplification was conducted to target the hypervariable V3–V4 regions of the 16S rRNA bacterial genes. Specific primers with barcodes and high-efficiency enzymes were used to perform PCR. The PCR primers were: forward 341F: CCTAYGGGRBGCASCAG; reverse 806R: GGACTACNNGGGTATCTAAT. The PCR products of 450–500 bp were collected with 2% agarose gel electrophoresis. To build library, same amount of PCR products from each sample is pooled, end-repaired, A-tailed and further ligated with Illumina adapters. The library QC was performed with Qubit and real-time PCR for quantification and with bioanalyzer to check the insert size distribution. Libraries were sequenced on a paired-end Illumina platform to generate 250 bp paired-end raw reads. The raw sequencing data are available in Zenodo (https://doi.org/10.5281/zenodo.7920752; Publication date: May 10, 2023).

### 2.4. Bioinformatic analysis and statistics

We used the R platform for statistical analysis and for the data processing. We applied the DADA2 workflow (28) to infer the Amplicon Sequence Variants (ASVs) and for the taxonomic assignments.

In brief, we first filtered and trimmed raw sequencing reads in order to remove low quality bases and adapter contamination. Then, we removed identical reads. Moreover, the reads were denoised, merged filtered to remove artifacts (PCR, and PhiX related chimeras). We obtained the ASVs quantifications and assigned taxonomy annotations (including the Species level) using the SILVA database of non-redundant sequences (version: v138, nr99) (29). The data were structured in objects including the ASVs quantifications, the taxonomy annotations, the sample group data and the phylogenetic tree using the phyloseq and the APE packages (30, 31). Finally, based on the initial DNA concentration, we removed possible contaminant ASVs by using the “prevalence” method of the decontam package (32).

Downstream analyses were performed using the MicrobiomeAnalystR package (33, 34) and included data normalization, measures of diversity and differential abundance estimation.

Briefly, we normalized the ASV counts based on their abundance (low count filter: for any ASV to be retained, at least 20% of its values should contain at least 4 counts) and variance (low variance filter: based on Inter-quantile range ± 10%). This, because ASVs with small counts (in few samples) could represent sequencing errors. Moreover, ASVs that are closely constant in all samples could be excluded from the comparative analyses. Finally, we used the total sum scaling in order to bring all the samples to the same scale.

We evaluated the alpha-diversity by calculating the Abundance-based coverage estimator (ACE) and a nonparametric estimator of species richness (Chao1) indices and by the Fisher metrics (to consider both richness and eveness). The degree to which the species composition changes between the two groups (the beta-diversity) was obtained by PCoA (Principal Coordinates Analysis) of the distances calculated with the un-weighted UniFrac method and the statistical significance assessed by the PERMANOVA test.

We also used the rarefaction curves to evaluate whether the samples were sufficiently sampled and sequenced to represent their species richness. We assessed the statistical significance of comparisons between the two groups of samples under study by using the Mann–Whitney test.

The differential abundance was assessed by the LEfSe (Linear Discriminant Analysis Effect Size) algorithm (35) for biomarker discovery and interpretation of metagenomics data. It involves the Kruskal-Wallis rank sum test to identify features (e.g., Species or Genera) with significant differential abundance in the two groups, followed by linear discriminant analysis (LDA) to evaluate the relevance (the effect size) of the selected features. Different abundant features were considered if the FDR adjusted *value of p* was less than or equal to 0.05 and if the Log LDA was greater than or less than 0.5. We used Phylogenetic Investigation of Communities by Reconstruction of Unobserved States (PICRUSt) to assess the metabolic potential of microbial communities (KEGG pathways). In this analysis, we started from the ASVs belonging to the significant genera obtained by LefSE algorithm.

## 3. Results

### 3.1. Cohort characteristics

Anthropometric characteristics and eating habits of the children enrolled in this study are shown in Table 1. No significant differences in individuals’ gender (Chi-square 0.6748; *p* = 0.411399). anthropometric characteristics such as height, weight, BMI and the waist/height ratio were observed (*p* > 0.05). Similarly, no significant differences in all diet components analyzed in A and S groups were found (Table 1).

**Table 1:** Anthropometric characteristics and eating habits of Active (A) and Sedentary (S) children.

	Total	Active (A)	Sedentary (S)
Gender M/F	69/45	33/18	36/27
Age (years)	8–10	8–10	8–10
*Anthropometric data*			
Height (cm)	142.8 ± 7.5	143.5 ± 8.0	142.2 ± 7.0
Weight (kg)	38.7 ± 9.8	38.2 ± 9.3	39.2 ± 10.2
BMI (kg/m^2^)	18.9 ± 3.8	18.5 ± 3.7	19.2 ± 3.9
Waist/Height (cm)	0.5 ± 0.06	0.45 ± 0.05	0.5 ± 0.06

Eating habits (Average daily intake)	Active	Sedentary
Calories (kcal)	1464.7 ± 284.2	1491.1 ± 281.1
Carbohydrates (%)	48.1 ± 5.8	48.9 ± 6.2
Carbohydrates (g)	188.2 ± 43.8	194.0 ± 43.6
Starch (g)	75.8 ± 27.3	70.8 ± 27.0
Oligosaccharides (g)	50.7 ± 19.6	55.9 ± 18.6
Oligosaccharides/Carbohydrates (%)	26.7 ± 7.1	29.1 ± 8.7
Lipids (%)	36.1 ± 5.8	36.1 ± 5.5
Lipids (g)	58.5 ± 13.5	59.7 ± 13.7
Saturated fatty acids (g)	14.2 ± 4.6	15.3 ± 4.7
Monosaturated fatty acids (g)	18.7 ± 5.6	18.7 ± 6.0
Polysaturated fatty acids (g)	4.8 ± 1.4	5.0 ± 1.9
Saturated/ fatty acids (%)	37.4 ± 6.5	39.5 ± 6.3
Cholesterol (mg)	135.6 ± 64.7	144.9 ± 78.9
Proteins (%)	15.5 ± 2.0	15.0 ± 2.2
Proteins (g)	56.9 ± 13.8	55.9 ± 13.7
Animal proteins/Proteins (%)	70.6 ± 9.3	72.6 ± 14.1
Vegetal proteins/Proteins (%)	29.4 ± 9.3	27.4 ± 14.2
Total fiber/1,000 (kcal)	7.8 ± 3.2	6.8 ± 2.2
Total fiber (g)	11.7 ± 6.2	10.0 ± 3.3

### 3.2. Sequencing reads processing and taxonomic assignments

The Illumina sequencing of the hypervariable V3–V4 regions of the 16S rRNA bacterial genes generated 2 × 250 bp paired-end reads. On average, we obtained 169.124 reads per-sample (Supplementary Figure S1A). Overall, the percentage of bases with quality scores above 20 and 30 (Q20 and Q30, respectively) was of 96.45 and 91.22, respectively (Supplementary Figures S1B,C). The percentage of GC nucleotides was of 52.03 (Supplementary Figure S1D). The set of reads was used to run the DADA2 workflow including the filtering and trimming (median = 169.02), denoising of forward (median = 165.00) and reverse (median = 164.80) reads; merging (median = 149.27) and chimeric reads removal (median = 114.43; Supplementary Figure S1E). After merging, the median length of reads was of 424 bp. Overall, starting from filtered reads, we obtained a merging rate of 88.38% and a final rate of read processing (non-chimera over merged reads) of 67.62% (Supplementary Figure S1F). Details on read processing are reported in Supplementary Table S1.

For taxonomic assignments we used the SILVA database of non-redundant sequences (version: v138, nr99). Overall, we could identify a total of 14.197 taxa (ASVs) that were annotated to the seven taxonomic ranks as follows. All the ASVs were taxonomically assigned to the kingdom of bacteria. The 98.06% of the ASVs was annotated at the phylum level (43 phyla), the 96.97% at the class level (94 classes), the 94.93% at the order level (195 orders), the 88.43% at the family level (231 families), the 81.04% at the genus level (404 genera) and the 4.86% was annotated up to the species level (257 species; Supplementary Figure S2A). From the initial set of annotated ASVs, we discarded a total of 68 taxa as possible contaminants. Moreover, as described in Methods we removed low abundant and low variable ASVs to obtain the final set of 472 ASVs that was normalized and used for downstream analyses.

### 3.3. Diversity estimates

**Figure 1:**
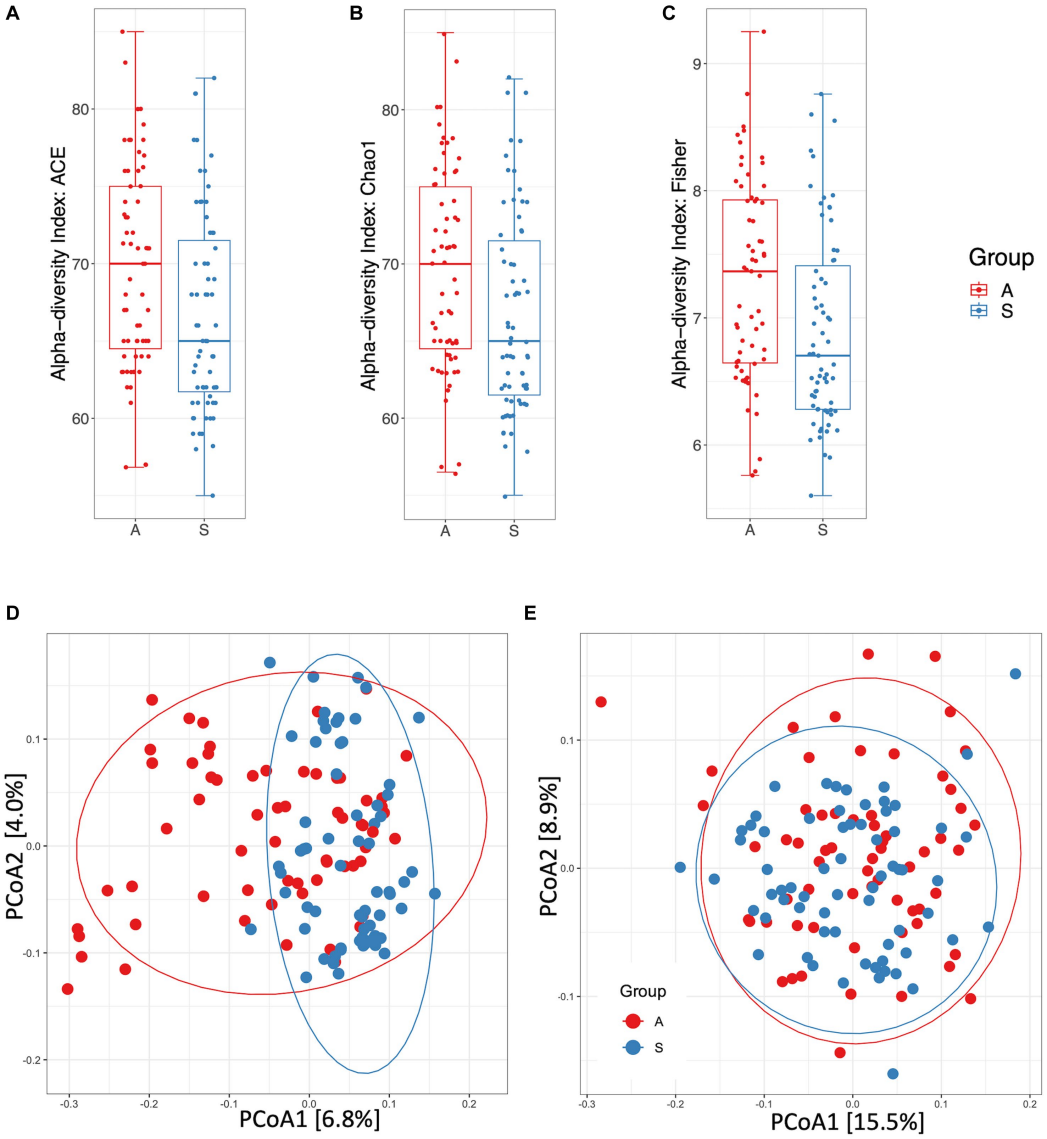
Diversity and distance measures between active (A) and sedentary (S). **(A)** Alpha-diversity measured by ACE index (*p* = 0.0099). **(B)** Alpha-diversity measured by Chao1 index (*p* = 0.0097). **(C)** Alpha-diversity measured by Fisher index (*p* = 0.0039). **(D)** Principal Coordinates Analysis plot of beta-diversity index measured by unweighted UniFrac distances (*p* = 0.001). **(E)** Principal Coordinates Analysis plot of beta-diversity index measured by weighted UniFrac distances (*p* = 0.001). (**A)**, B, C, F: Mann–Whitney test. (**D)**, E: PERMANOVA test.

At the genus level, alpha-diversity estimates were significantly different between the two groups, A and S. Indeed, ACE (Figure 1A), Chao1 (Figure 1B) and Fisher (Figure 1C) indices showed *p* < 0.01 (Mann–Whitney test). The beta-diversity analysis, as measured by unweighted UniFrac distances, showed a significantly different microbial composition between the two groups (*r*^2^ = 0.026, *p* = 0.001; Figure 1D), the diversity was also confirmed by using the weighted UniFrac distance metric (*r*^2^ = 0.018, *p* = 0.001; Figure 1E). As previously reported (36), unweighted and weighted UniFrac distance measures can be considered as quality-based and quantity-based indexes, respectively. Indeed, we can assess that the observed variation between the two groups was due to the different taxa abundances and to the types of taxa in their microbiome. Moreover, the rarefaction analysis clearly evidenced the capacity to capture the species richness from the results of sampling and sequencing in both groups without any statistically significant difference (*p* = 0.084; Supplementary Figure S2B).

### 3.4. Abundance estimates

We evaluated and compared the taxa abundance in the final set of 472 filtered and normalized ASVs. Overall, we identified 8 phyla, 12 classes, 30 orders, 46 families, 84 genera and 96 species.

**Figure 2:**
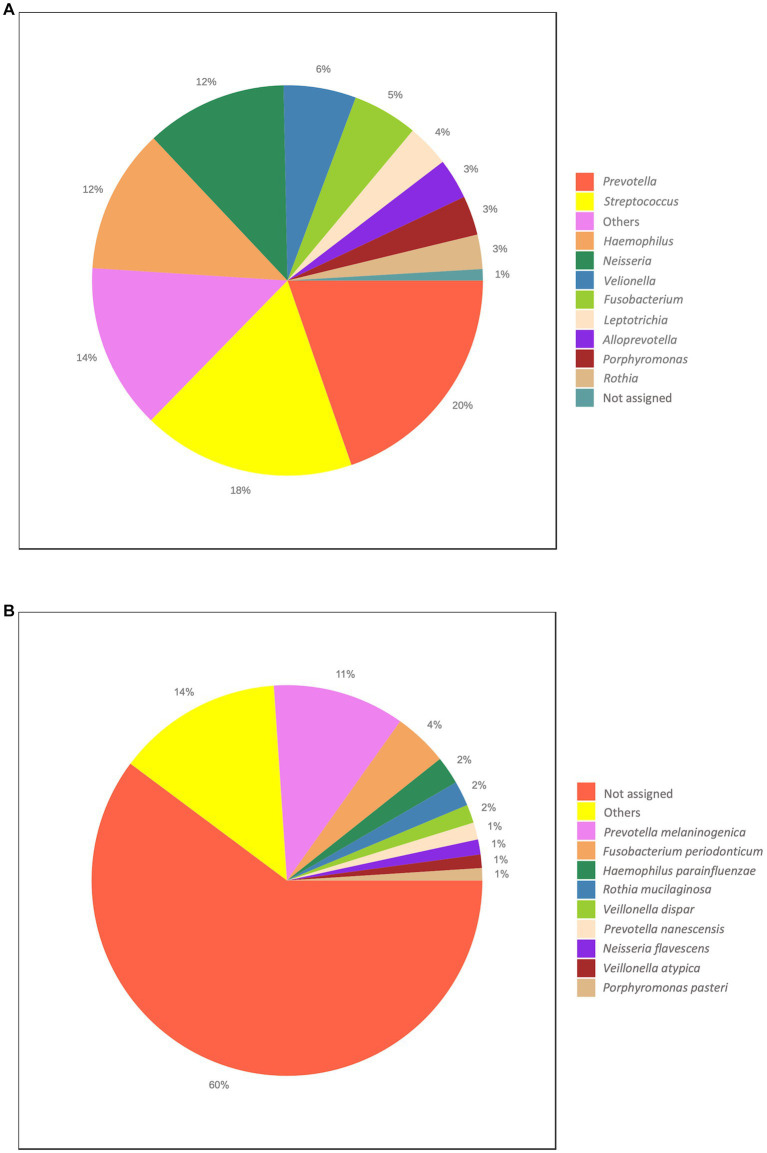
Overall taxonomic distribution. The Figure reports the overall abundance of the identified taxa. **(A)** Genus level. **(B)** Species level. Each plot shows the top ten abundant taxa.

At the phylum level, on average, the most abundant bacteria were Firmicutes, Bacteroides and Proteobacteria accounting for the 32.08, 26.97% and the 25.58% of the taxa, respectively (Supplementary Figure S3A). The most represented classes were Bacteroides (26.97%), Gammaproteobacteria (25.58%) and Bacilli (21.36%; Supplementary Figure S3B). The most prevalent orders were Bacteroides (26.63%), Lactobacillales (19.58%), Pasteurellales (12.88%) and Burkholderiales (12.12%; Supplementary Figure S3C). Among the most abundant families, we found Prevotellaceae (23.07%), Streptococcaceae (17.66%), Pasteurellaceae (12.88%) and Neisseriaceae (11.77%; Supplementary Figure S3D). At the genus level, we found the *Prevotella* (19.74%), *Streptococcus* (17.66%), *Haemophilus* (11.87%), *Neisseria* (11.57%) and *Veillonella* (6.06%; Figure 2A). Finally, the top abundant species that we were able to classify were *Prevotella melaninogenica* (10.86%), *Fusobacterium periodonticum* (4.44%), *Haemophilus parainfluenzae* (2.27%), *Rothia mucilaginosa* (2.10%) and *Veillonella dispar* (1.55%; Figure 2B).

### 3.5. Differential abundance estimates

As described in Methods, we used the LEfSe algorithm to perform the differential abundance analysis and to identify the taxa that could explain the differences between the two groups A and S. We set the threshold at 5% and the Log LDA cutoff at ± 0.5. Interestingly, using these very stringent criteria, we found *Coriobacteriaceae* family as more abundant in the A compared to the S group (LDA score = −3.17; FDR = 0.021).

**Figure 3:**
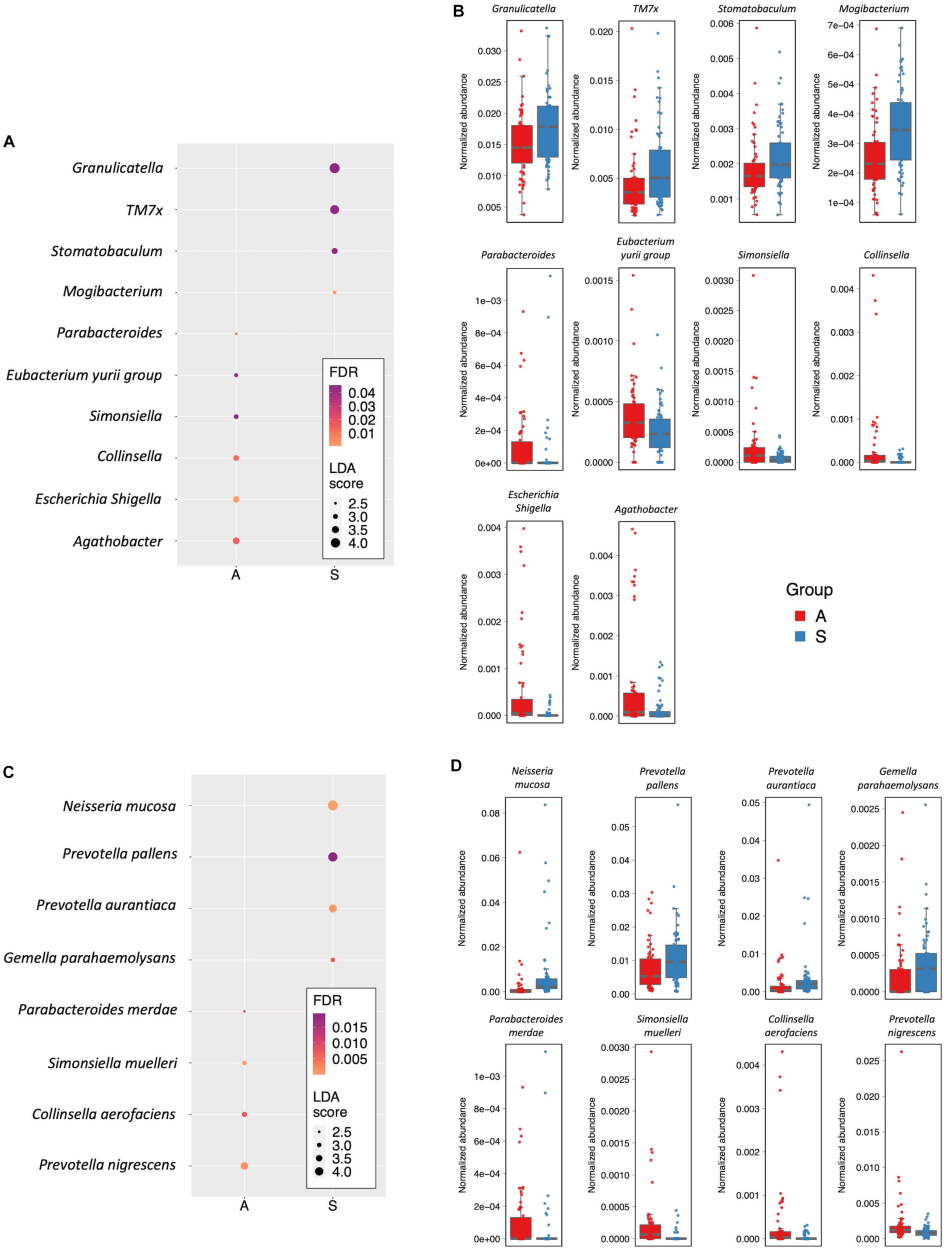
Differently abundant taxa. **(A)** Dot plot showing the differently abundant genera. **(B)** Box plots showing the Normalized abundance levels of genera reported in panel A. **(C)** Dot plot reporting the differentially abundant species. **(D)** Box plots showing the normalized abundance levels of species reported in panel C. In panels A and C, the dot size is proportional to the score of the LDA algorithm. The dot graduation color is proportional to the significance level as determined by FDR adjustment of Kruskal-Wallis rank sum test *p* values. A: Active; S: Sedentary.

Further, at the genus level, we found that ten genera were responsible for the differences between the two groups. In particular, *Agathobacter* (LDA score = −3.40; FDR = 0.015), *Escherichia*–*Shigella* (LDA score = −3.37; FDR = 7.68×10-04), *Collinsella* (LDA score = −3.17; FDR = 0.012), *Simonsiella* (LDA score = −2.95; FDR = 0.044), *Eubacterium*-*yurii* group (LDA score = −2.79; FDR = 0.041) and *Parabacteroides* (LDA score = −2.43; FDR = 0.015) were more abundant in the A group. On the contrary, *Mogibacterium* (LDA score = 2.71; FDR = 9.21×10–04), *Stomatobaculum* (LDA score = 3.24; FDR = 0.44), TM7× (also known as *Nanosynbacter lyticus*, LDA score = 3.90; FDR = 0.045) and *Granulicatella* (LDA score = 4.14; FDR = 0.045) were more abundant genera in the S group (Figures 3A,B; Supplementary Table S2). Eight species showed significant differences in the LEfSe analysis. Indeed, *Prevotella nigrescens* (LDA score = −3.76; FDR = 1.5 × 10–03), *Collinsella aerofaciens* (LDA score = −3.17; FDR = 7.45 × 10–03), *Simonsiella muelleri* (LDA score = −2.96; FDR = 2.76 × 10–05), *Parabacteroides merdae* (LDA score = −2.43; FDR = 1.3 × 10–02), were the most represented species in the A group. Conversely, *Gemella parahaemolysans*, *Prevotella aurantiaca* (LDA score = −3.9; FDR = 5.27 × 10–04), *Prevotella pallens* (LDA score = 4.23; FDR = 1.93 × 10–02), *Neisseria mucosa* (LDA score = 4.43; FDR = 1.31 × 10–02) were more abundant species in the S group (LDA score = 2.94; FDR = 7.45×10-03; Supplementary Table S3; Figures 3C,D).

### 3.6. Metabolic pathways reconstruction

**Figure 4:**
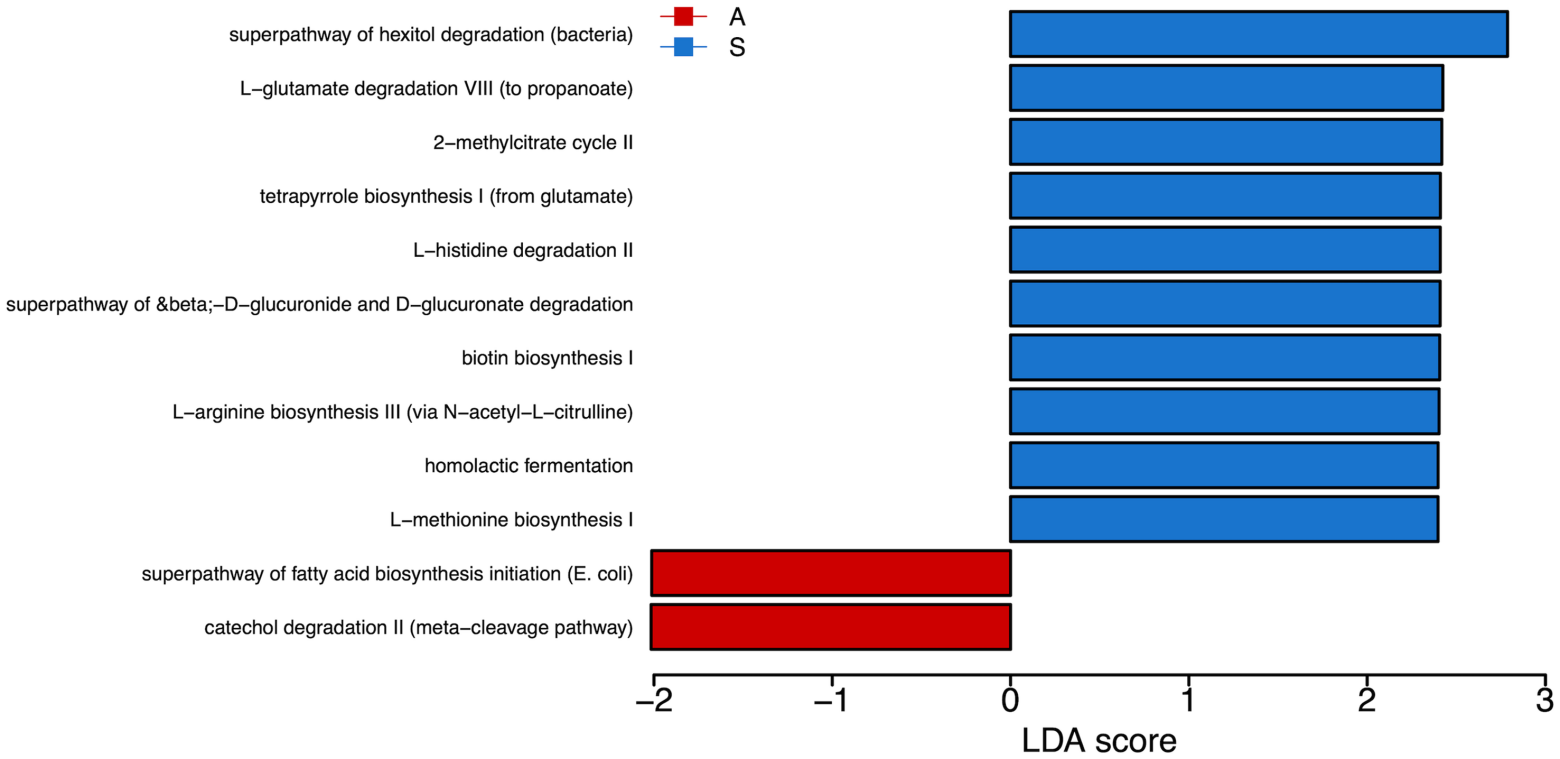
Metabolic pathways reconstruction. We used Phylogenetic Inves-tigation of Communities by Reconstruction of Unobserved States (PICRUSt) to predict the activity of metabolic pathways (KEGG) starting from the significant genera obtained by LefSE analysis. In red are the pathways enriched in the A group. In blue are the pathways enriched in the S group. A: Active; S: Sedentary.

PICRUSt analysis highlighted the predominance of super pathway of hexitol degradation, L-glutamate degradation VII (to propionate), 2-methylcitrate cycle II, tetrapyrrole biosynthesis I, L-histidine degradation II, superpathway of beta-D-gluconide and D-gluconate degradation, biotin biosynthesis I, and L-arginine biosynthesis pathways activation in saliva from S compared to A children. Conversely, we found a prevalence of superpathway of fatty acid biosynthesis initiation (*E. coli*) and catechol degradation II (meta-cleavage pathway) in saliva from A respect to S children (Figure 4).

## 4. Discussion

The aim of the study was to evaluate the effects of Active or Sedentary lifestyle on saliva microbiota composition in Italian schoolchildren living in the outskirts of Turin. The participants were classified as Active (A) or Sedentary (S) according to the cut-off score of 2.75 for PAQ-C-It (27). We evidenced an enrichment of several genera, such as *Agathobacter*, *Collinsella*, *Simonsiella*, and *Parabacteroides* in the children’s saliva from A compared to S group. Among these, four species were differentially represented: *Prevotella nigrescens*, *Collinsella aerofaciens*, *Simonsiella muelleri*, and *Parabacteroides merdae*. Increased abundance of *Agathobacter* and *Prevotella* at both genus and species levels, was reported in GM of cross-country and marathon athletes; although an inverse correlation was found for Prevotella and sucrose intake and a positive correlation for Agathobacter and dietary fiber content (37), we did not find statistically significant differences in all dietary components, including fiber content in A compared to S children. *Parabacteroides* are involved in host health promotion by regulating different pathways including inflammation, obesity and cancer prevention (38). Moreover, recent data suggest an anti-seizure and anti-cancer functions for *Parabacteroides merdae* (39, 40) and increased abundance have been also found in the GM of centenarians living in East China (41). As no differences in BMI were found between A or S children belonging to our cohort, we speculate that the prevalence of *Parabacteroides merdae* in saliva of group A children could be associated to the higher level of daily Physical Activity Amounts (PAA) when compared to group S. Furthermore, the increased abundance of *Parabacteroides* in group A resulted in the enrichment of the superpathway of fatty acid biosynthesis initiation that we found by metabolic pathway reconstruction. Of note, *Parabacteroides* are also involved in regulating different processes as carbohydrates metabolism and metabolites secretion, including Short Chain Fatty Acids (SCFAs) (42, 43) Among them, acetate, propionate, and butyrate are the main metabolites produced by several anaerobic bacteria from the fermentation of complex starch and dietary fibers. The available mechanistic data strongly suggest that SCFAs exert their powerful anti-inflammatory, antitumorigenic and even antimicrobial effects in the preventing gastro-intestinal dysfunction, obesity and type 2 diabetes mellitus (44, 45). In line with these evidences, several studies several studies conducting in patients with type 1 and type 2 diabetes, liver cirrhosis, inflammatory bowel disorders (IBD) and atherosclerosis have shown a reduction in the abundance of SCFA-producing bacteria gut (46, 47). Gut microbiota of athletes have an enriched profile of SCFAs, previously associated to a healthier status and a lean phenotype (44, 48). In skeletal muscle, SCFAs can be oxidized, incorporated into glucose *via* gluconeogenesis or increase the bioavailability of glucose, glycogen and fatty acids during exercise (49). Similarly, increased abundance in GM of taxa as *Firmicutes* and *Feacalibacterium prausnitzii* together with *Akkermansia,* producing butyrate, have been associated to exercise in athletes and non-athletes’ controls with improvement in lipid oxidation, healthier profile and reduced risk for obesity and metabolic diseases, independently from body composition and diet (50–53). Further, similarly to our results, the association of a healthier profile with a reduction in *Bacteroides* species together with an increase in *R. hominis*, *A. Muciniphyla* and *F. prausnitzii* species have been described in GM from Active compared to Sedentary adults (54–56).

In group S we found an increased abundance of *Gemella parahemolysan*, *Prevotella aurantiaca*, *Prevotella pallens* and *Neisseria mucosa* species and of the *TM7x* genus as compared to group A. Notably, previous studies reported the abundance of *Neisseria mucosa* as sixfold higher in obese adolescents compared to normal-weight controls (57). Suggesting that although the Sedentary children are normal-weigh, they present a predictive marker linked to obesity. Moreover, *Prevotella* species, habitually present in the oral microbiome, have constant and direct access to the gastrointestinal tract *via* saliva swallowing. Here, they could act as commensals but also as potentially harmful agents (58). Furhermore, the group S showed an increased abundance of the genus *TM7x* (also known as *Nanosynbacter lyticus*) which is an obligate epibiont parasite of the bacteria *Actinomyces odontolyticus* (not significantly enriched in our data) (59, 60). *TM7x* have been previously associated to different human inflammatory mucosal diseases such as the periodontitis (61). Moreover, *TM7x* have been considered as biomarker of active disease in patients with ulcerative colitis (62).

In group S, the metabolic pathway reconstruction highlighted the enrichment of L-glutamate degradation and L-arginine biosynthesis pathways. Interestingly, the dysregulation of L-glutamate and L-glutamine pathways have been associated with poor survival in colon cancer patients (63, 64). L-glutamate signaling triggers oxidative and nitrosative stress pathways which are essential for the production of ROS that can induce the activation of oncogenes ensuring the survival of colon cancer cells (64).

Conversely, the analysis performed with PICRuST on A children’s saliva revealed an abundance of fatty acid biosynthesis and catechol degradation pathways, in line with previous reports (65). The catecholamines are catabolic intermediates of various aromatic compounds, which contribute to Acetyl-CoA production. Acetyl-CoA, is also crucial for the cross-talk between multiple biological processes including, energy storage, membrane biosynthesis, and generation of signaling molecules that are produced in response to physiological cell processes (66, 67). Consequently, dysregulation of fatty acid synthesis can induce or promote disease development (68, 69).

In conclusion, Our results showed that saliva from active children had an enrichment of species and genera mainly associated with a healthier profile. On the contrary, the genera and the species enriched in the saliva from sedentary group could be linked non-communicable diseases. Nevertheless, our indirect observations need to be clarified by further (and possibly larger) studies aimed at understanding how an active lifestyle can modulate the composition of both oral and gut microbiota. Moreover, the minimum volume of physical exercise required to determine changes in oral microbiota composition remains to be assessed.

## Data availability statement

The datasets presented in this study can be found in online repositories. The names of the repository/repositories and accession number(s) can be found in the article/Supplementary material.

## Ethics statement

The studies involving human participants were reviewed and approved by Ethics committee on human research of the University of Turin (9 March 2020: Protocol #134691) and Naples (17 January 2020: Protocol #376/19) approved the study. Written informed consent to participate in this study was provided by the participants’ legal guardian/next of kin.

## Author contributions

AM, SO, and PBu: conceptualization. DV, DP, CC, and AC: methodology. VL and MC: software, validation, and formal analysis. PBr and CL: investigation and resources. AM, DV, and VL: data curation and writing—original draft preparation. AM, SO, MC, and PBu: writing—review and editing. PBu: visualization and supervision. AM, SO, FS, and PBu: project administration and funding acquisition. All authors contributed to the article and approved the submitted version.

## Funding

This study was funded by the grant PRIN 2017_Prot.2017RS5M44 to PBu, AM, SO, and FS.

## Conflict of interest

The authors declare that the research was conducted in the absence of any commercial or financial relationships that could be construed as a potential conflict of interest.

The reviewer GC declared a past co-authorship with the author CC to the handling editor.

## Publisher’s note

All claims expressed in this article are solely those of the authors and do not necessarily represent those of their affiliated organizations, or those of the publisher, the editors and the reviewers. Any product that may be evaluated in this article, or claim that may be made by its manufacturer, is not guaranteed or endorsed by the publisher.
