# Supplementary figures and images for: Impact of active lifestyle on the primary school children saliva microbiota composition
Source: Front Nutr. 2023 Aug 10;10:1226891. doi: 10.3389/fnut.2023.1226891 (PMC10476528; doi:10.3389/fnut.2023.1226891)

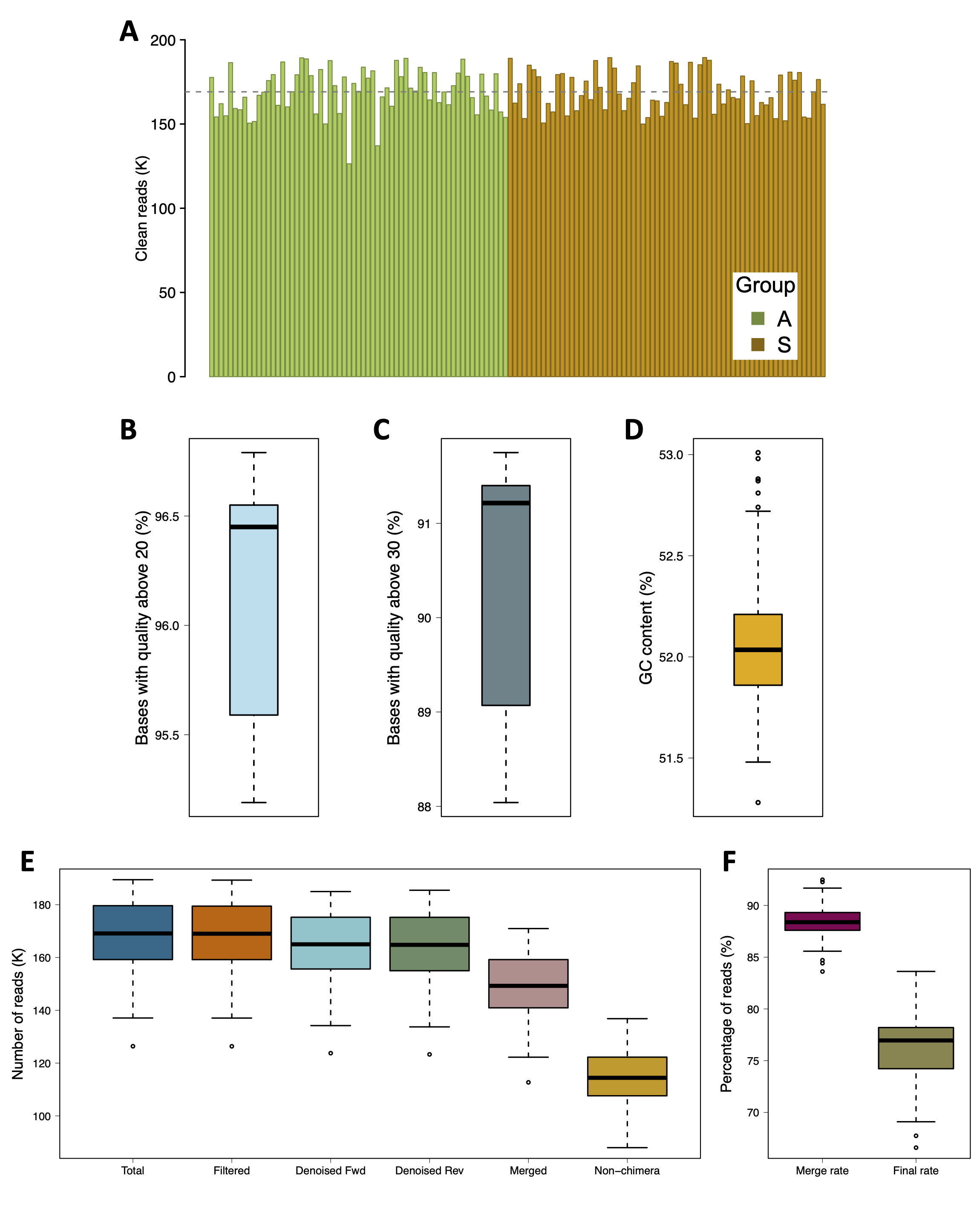

Supplement: Supplementary file 1 [file Image_1.TIFF]

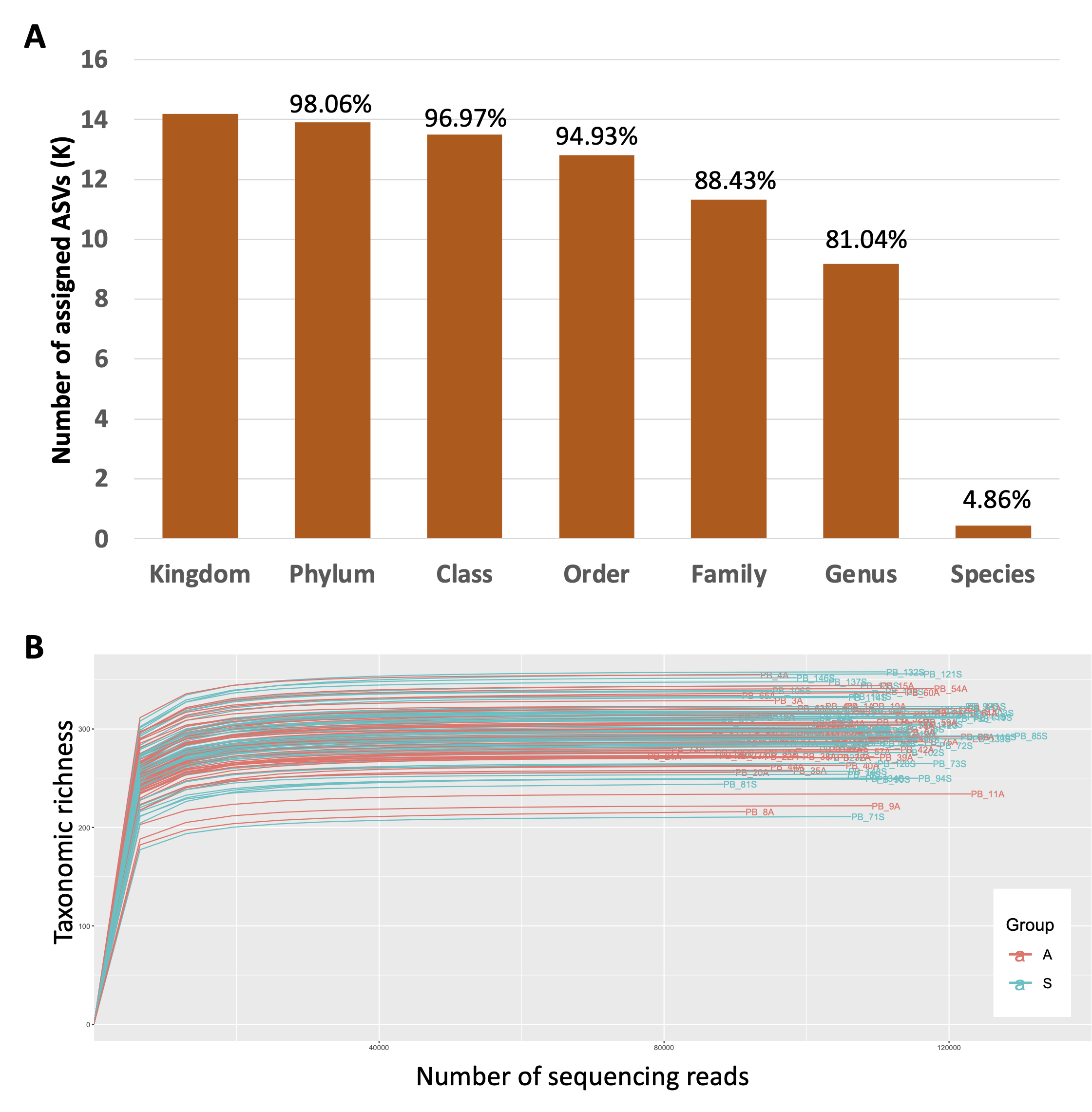

Supplement: Supplementary file 2 [file Image_2.TIFF]

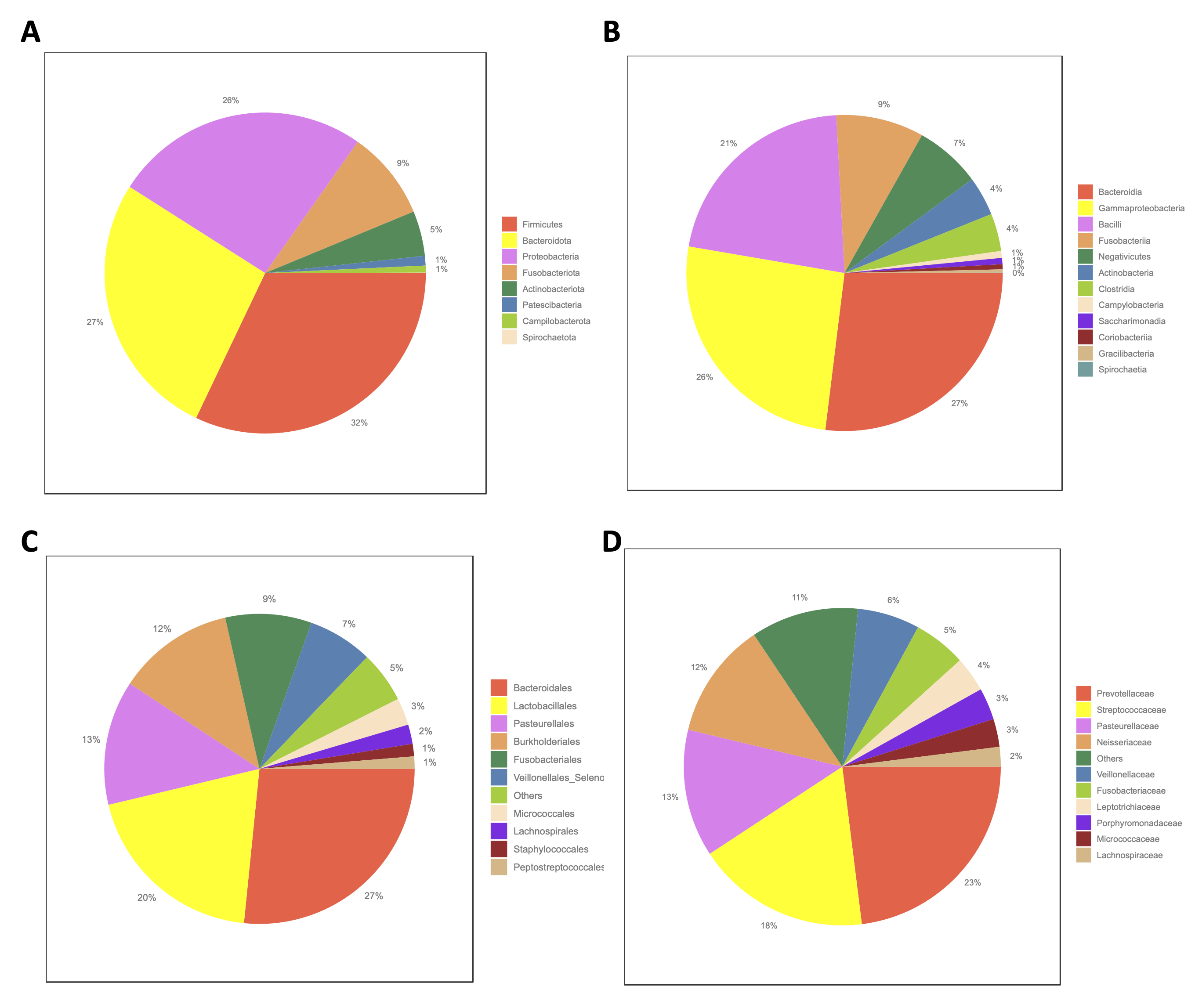

Supplement: Supplementary file 3 [file Image_3.TIFF]
